# Hypoxia Tolerant Species: The Wisdom of Nature Translated into Targets for Stroke Therapy

**DOI:** 10.3390/ijms222011131

**Published:** 2021-10-15

**Authors:** Carmen del Río, Joan Montaner

**Affiliations:** 1 Institute of Biomedicine of Seville (IBiS), Hospital Universitario Virgen del Rocío, CSIC, Universidad de Sevilla, 41013 Seville, Spain; jmontaner-ibis@us.es; 2 Department of Neurology, Hospital Universitario Virgen Macarena, 41009 Seville, Spain

**Keywords:** stroke, neuroprotection, hypoxia tolerance, stroke therapy

## Abstract

Human neurons rapidly die after ischemia and current therapies for stroke management are limited to restoration of blood flow to prevent further brain damage. Thrombolytics and mechanical thrombectomy are the available reperfusion treatments, but most of the patients remain untreated. Neuroprotective therapies focused on treating the pathogenic cascade of the disease have widely failed. However, many animal species demonstrate that neurons can survive the lack of oxygen for extended periods of time. Here, we reviewed the physiological and molecular pathways inherent to tolerant species that have been described to contribute to hypoxia tolerance. Among them, Foxo3 and Eif5A were reported to mediate anoxic survival in *Drosophila* and Caenorhabditis elegans, respectively, and those results were confirmed in experimental models of stroke. In humans however, the multiple mechanisms involved in brain cell death after a stroke causes translation difficulties to arise making necessary a timely and coordinated control of the pathological changes. We propose here that, if we were able to plagiarize such natural hypoxia tolerance through drugs combined in a pharmacological cocktail it would open new therapeutic opportunities for stroke and likely, for other hypoxic conditions.

## 1. Introduction

Stroke remains as a leading cause of death and disability worldwide. Stroke is caused by the focal and sustained disruption of blood supply (ischemia) to the brain, resulting in oxygen and nutrient deprivation. Oxygen is the final acceptor at the electron transport chain during aerobic respiration, which is essential for efficient energy production so tolerating hypoxia is not an easy road for most animal species. In fact, our brain is especially sensitive to ischemia due to high oxygen and nutrient requirements and the inability to store metabolic products [1] so human neurons die shortly after ischemia.

Despite the social and financial burden of stroke, calculated in 2015 by the Stroke Alliance for Europe as €45 billion just in the European Union, endovascular thrombectomy and intravenous thrombolysis are the only approved medical therapy for ischemic stroke. Alteplase (recombinant tissue plasminogen activator [rtPA]) is a thrombolytic agent that assist in the formation of plasmin initiating fibrinolysis to restore blood flow administered to eligible stroke patients within 4.5 h of symptom onset. A second thrombolytic agent, Tenecteplase, is now recommended by the current guidelines over intravenous thrombolysis with alteplase for patients with acute ischemic stroke of <4.5 h duration with large vessel occlusion for whom intravenous thrombolysis is considered [2]. Overall, reperfusion therapy has some use limitations, such as the risk of hemorrhagic events, moderate recanalization rates and a narrow therapeutic window that restricts to 15% the number of patients that can be treated [3].

There are fascinating examples in nature of animals exceptionally tolerant to the lack of oxygen which cope with extreme environmental conditions that would be lethal to humans, such as pond turtles and crucian carps, that can survive anoxia under ice-covered lakes and ponds for several months. In such conditions temperatures are close to 0 °C and clearly contribute to the extreme tolerance in both species, but even at higher temperatures they can still survive anoxia for at least a day or two [4]. Moreover, some mammals are also able to cope with reduced oxygen availability for relatively long periods without injury, such as diving mammals and the naked mole rat [5].

**Figure 1:**
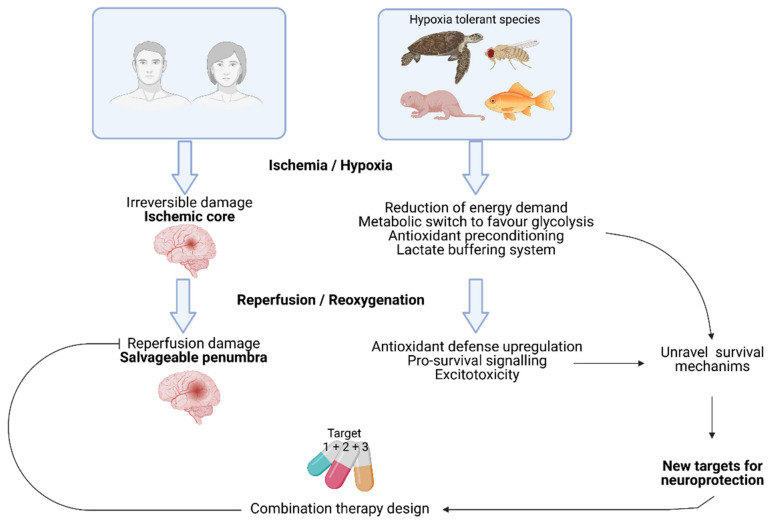
Human brain quickly undergoes irreversible cell death during ischemia. However, the surrounding penumbra is a salvageable tissue. Hypoxia tolerant brains have evolved different complementary mechanisms to deal with the lack of oxygen for prolonged periods of time that are not fully understood. A better knowledge of the survival mechanisms used by these animal species at the molecular level can provide new targets for neuroprotection that combined into pharmacological cocktails might help in the recovery of damaged brain cells.

How tolerant species keep their neurons alive after days of hypoxia, an astonishing natural capacity acquired throughout evolution, is not fully understood. Results from animal studies suggest that neuroprotective agents can prevent brain damage and neurological deficits after experimental brain ischemia. In this sense, different neuroprotective approaches targeting known molecular pathways used by tolerant animals have resulted in positive outcomes in preclinical stroke models. Unfortunately, all drugs tested in preclinical studies have systematically failed to translate in human ischemia. Understanding the mechanisms that operate simultaneously in tolerant animals to grant them survive hypoxia without neurological damage may yield different insights to propose new neuroprotective combination therapies for stroke that overcome the failure of individual treatments (Figure 1).

## 2. Stroke Pathophysiology: Molecular Consequences of Ischemia

Stroke is a complex disease which involves many pathological mechanisms. Cell death occurs within minutes of ischemia and the brain region that has already progressed to irreversible injury is called the ischemic core. The surrounding area, defined as the ischemic penumbra, undergoes functional impairment but is structurally intact so cell viability can be maintained if reperfusion is achieved rapidly [6]. Faster and more complete arterial reperfusion correlates with superior treatment efficacy, but highly effective reperfusion is not enough for functional independence in 50% of the patients [7].

**Figure 2:**
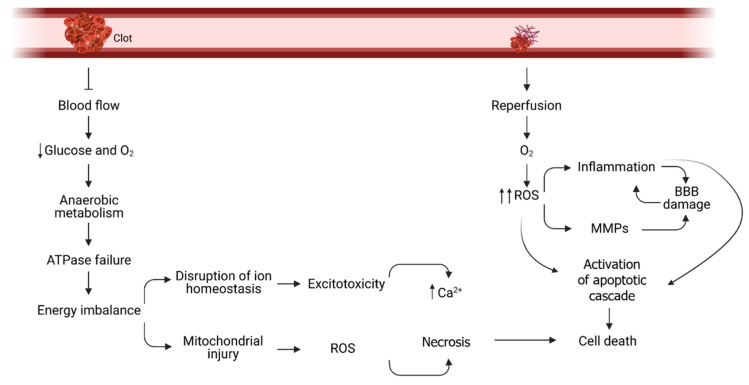
Ischemic cascade during stroke. Hypoperfusion of a brain area during stroke causes a rapid energy imbalance. Low ATP levels cannot maintain ionic homeostasis and mitochondrial function, which ultimately lead to cell death in the ischemic core. After reperfusion, overproduction of Reactive Oxygen Species (ROS) triggers post-ischemic inflammation and the leakage of the Blood Brain Barrier (BBB) that amplifies cytotoxicity at the penumbra. Abbreviations: matrix metalloproteinase (MMPs).

The insufficient supply of oxygen and glucose to the brain cells generates a complex cascade of pathological events (Figure 2). First, the mitochondrial oxidative phosphorylation is disrupted and, therefore, ATP synthesis is decreased and energy stores quickly deplete. Anaerobic glycolysis is not enough to cover the cellular needs and the accumulation of lactate as an end-product triggers tissue acidosis. The immediate consequence of the energy shortage is the failure of ATPases to work properly and consequently ion imbalance. At this point, neurons depolarize and the entrance of calcium results in the activation of catabolic and cell death pathways but also in the release of excessive amounts of excitatory neurotransmitters [8]. Moreover, dysregulation of Na^+^/K^+^ exchange increases intracellular sodium concentration that can lead to cellular edema [6]. Glutamate, the main excitatory neurotransmitter, further exacerbates Ca^2+^, K^+^ and Na^+^ influx and the downstream signaling cascade. Calcium overload activates calcium-dependent enzymes (lipases, phosphatases, and endonucleases) that cause membrane disruption, DNA damage and Reactive Oxygen Species (ROS) production [9]. The extent and mechanism of cell death is dependent on the duration and grade of blood flow reduction. At the ischemic core, cells quickly undergo necrosis and the damage spread towards the periphery causing delayed cell death by apoptosis at the penumbra area [10]. Restoration of blood flow represents, paradoxically, another source of cell injury by providing oxygen that can be used for ROS production in a way that exceeds the capacity of the antioxidant defenses and directly damage lipids, proteins, nucleic acids, and carbohydrates, and participate in signalling pathways that can promote cell death programs [11]. ROS and Ca^2+^ accumulation promote the synthesis of pro-inflammatory mediators and endothelial cell adhesion molecules as well as activate matrix metalloproteinase (MMPs). This phenomenon disrupts the integrity of the vascular blood brain barrier (BBB) and helps immune cells to migrate and infiltrate the brain parenchyma, which contributes to the inflammatory response initiated a few hours after anoxia [1]. The mentioned pathophysiological processes appear at different time points after ischemia. However, they are not independent to each other, but overlap over the time and share molecular features.

## 3. The Failure of Treatment Translation

The administration of neuroprotectants early after ischemia would potentially lengthen the therapeutic window and increase the number of patients that can be treated with tPA in addition to assist in the recovery of brain cells not irreversibly damaged to reduce reperfusion injury [12]. Until the early beginning of the 21st century, more than a thousand drug candidates for stroke were reported, from which 114 were tested clinically in humans [13]. Since then, the number of interventional studies on stroke patients have significantly grown, but results have not been more encouraging. The failure to translate treatments that have previously worked in experimental models of cerebral ischemia to the clinic highlights the need to better adjust the design of the experiments to the reality of the disease, as proposed by current guidelines [14]. Possible reasons for the lack of translation were carefully reviewed not long ago and include the fact that neuroprotective drugs are usually administered before stroke or in a short time-window after the occlusion in animal models, which is not practical in human strokes [15]. In addition, not testing the new medications in combination with reperfusion therapies in clinical trials, differences in the evaluation of the therapeutic outcomes and the use of healthy and young animals in preclinical studies, together with the absence of publications reporting negative results also contribute to the gap of human translation [7].

The experimental model of choice as well as the treatment regimen used can also affect the results. The ischemic cascade is well described in laboratory animals, but it does not necessarily recapitulate human stroke, which might involve additional pathological processes. In this regard, a profound knowledge of the pathophysiology of the disease is necessary for a well-timed administration of the appropriate drug after stroke (antiinflammatory, antiexcitatory, antioxidant, etc.). Neuroprotective drugs are usually administered before or right after the reperfusion in animal studies and they have shown a limited therapeutic time window, which is not practical for clinical intervention [14]. Furthermore, drugs are preclinically tested in the daytime when rodents are inactive whereas clinical trials test neuroprotectants in active and awake patients. This fundamental mismatch in the circadian rhythm has been recently reported to influence experimental results and therefore, must be also considered for translation [16].

Recently, results from a phase III clinical trial on Nerinetide in acute ischemic stroke patients including participants undergoing both reperfusion therapies (rtPA and thrombectomy) have been published. Although primary and secondary outcomes failed to show significant differences compared to the control group (potentially caused by drug-drug interaction of tPA with Nerinetide being a target of plasmin for degradation), there was a treatment effect on patients that did not receive rtPA. A reduction in the mortality risk and infarction volume in these patients suggests that a neuroprotective therapy for stroke is possible [17].

Another consideration would be that the vast majority of neuroprotection trials are based on monotherapeutic strategies against a single target. This multi-target strategy seems promising, as it is the key to the neuroprotective efficacy of hypothermia in stroke [18] and because of the success shown in recent years in the use of therapeutic cocktails in the field of oncology. This is the case of the combination of resveratrol and valproate, which act in synergy to exert neuroprotection [19] and the strategy developed by a group in Maastrich using NOX-4 and NOS inhibitors, thus attacking nearby pathways in this case related to oxidative stress [20]. In addition, countering the inflammatory response after stroke by the combination of alpha-lipoic acid (a free radical scavenger) and Etanercept (a biologic TNF inhibitor) protected rat brain from experimental stroke more extensively than when the drugs were administered individually [21]. Taking into account the different moments of activation of the processes that occur in the ischemic cascade after stroke, the idea of the need for neuroprotective therapy combining different drugs that act on multiple targets does not seem unreasonable.

## 4. Tolerant Species: Mechanisms for Adaptation

Hypoxia tolerant animals may be able to live in different environmental conditions through metabolic adaptations that the human body cannot accomplish. Larger brains are usually associated with improved cognitive skills. However, the metabolic cost of extreme encephalization is high and may entail decreased tolerance to changes in environmental energy availability. Therefore, hypoxia sensitive animals use other mechanisms to deal with hypoxia such as phenotypic plasticity or migration. An increased metabolic rate and energetic trade-off reducing energy expenditure of other organs or functions are the two non-exclusive hypotheses proposed to meet the energetic cost of bigger brains. When comparing between 30 mormyrid fish species, relative brain size correlated with oxygen consumption rate and small-brained lineages tolerated hypoxia better than large-brained lineages. It seems the trade-off hypothesis can only explain moderate encephalization but extreme encephalization may probably need an increase in overall energy investment [22].

Literature agrees there is not just one responsible mechanism that can explain their anaerobic capacity. Instead, tolerance results from the complex and coordinated adaptations of molecular and physiological processes at the cellular, organ and whole-body levels. Numerous studies have described physiological changes taking place in these species, but many intrinsic molecular aspects remain unexplored.

It is worth mentioning that ischemic preconditioning renders the ischemia-susceptible human brain resistant to prolonged periods of ischemia. It has been shown in healthy adults that ischemic preconditioning improves dynamic cerebral autoregulation and regulates neuroprotective and inflammatory biomarkers in blood [23]. Remote ischemic preconditioning (RIC) consists of repeated episodes of ischemia/reperfusion in the limbs to provide protection to a remote organ such as the brain. RIC is well tolerated in stroke patients and may improve neurological outcomes [24]. This strategy can provide protection not only when applied before ischemia, but also when applied during or after the ischemic insult. The underlying mechanisms of how RIC’s protective effect is transmitted to a distant organ is not well understood but it is known that it improves cerebral perfusion [25] and humoral, neural and immune mechanisms are involved [26] and therefore represents a great example of protection by combining multiple targets.

There are common features underlying hypoxia tolerance such as the entrance into a reversible hypo-metabolic state to maintain a balanced low ATP levels, increased reliance on anaerobic energy metabolism, modulation of neurotransmission to be protected against excitotoxicity, enhanced antioxidant defense and efficient buffering to avoid acidosis. Table 1 summarizes the main mechanisms of hypoxia tolerance described for the best know animal species.

### 4.1. Regulation of Cellular Energetics

Reducing energy demand and upregulating anaerobic metabolism are critical requirement for anoxia tolerance. It has been reported that pond turtles enter a reversible coma to maintain a balance between ATP synthesis and consumption. Heart rate is decreased and the distinctive presence of high levels of glycogen in the brain provide a rapid source of energy until the glycogen stores in the liver can be processed [38]. The comparison between metabolites produced in the heart of pond turtles and mice exposed to either normoxia or hypoxia showed adjustments taking place in turtles to maintain organ function, including the ability to balance ATP levels and reduced succinate to fumarate ratio [39]. Moreover, proteomic analysis of the Western Painted Turtle showed that, under anoxia, several proteins were altered in the brain in order to initiate a response to maintain ATP level, reduce apoptosis and prevent neurodegeneration, maintain cognitive function and promote neurological repair [40]. Naked Mole Rats (NMRs) dramatically reduce the metabolic rate and remain active under long periods of hypoxia [46]. However, when exposed to total oxygen deprivation they quickly lose consciousness and switch to fructose as fuel to the brain under anaerobic conditions, being able to recover after 18 min in such circumstances [47]. Compared to mice, ischemia has a lower impact on mitochondrial function in NMR, with maintained mitochondria respiratory capacity and membrane integrity [55]. Insects also enter a hypometabolic comatose state within seconds of anoxia exposure. *Drosophila* and locust can recover from hours of anoxia, which is impressive compared to humans, and rely on anaerobic glycolysis to maintain low but constant ATP concentration [27,30]. In fact, a deleterious effect of acute anoxia on the Central Nervous System (CNS) of flies is questionable and *Drosophila* can be experimentally selected under hypoxic pressure to live and perpetuate in extremely low O_2_ environments [56].

Crucian carp is believed to survive winter exclusively using liver glycogen stores to maintain constant ATP, which is supported by the upregulation of key glycolytic enzymes during experimental anoxia [35]. The study of the heart proteome of the goldfish, which are regarded as a variant of the crucian carp, identified hypoxia-regulated enzymes involved in catalyzing reversible steps of the glycolysis/gluconeogenesis network, which allow the goldfish to recycle pyruvate [57]. Seals and whales rely on both large amounts of oxygen stored in haemoglobin and myoglobin and liver glycogen for energy production to perform their routine dives. However, at the end of the dive lactate accumulates secondary to oxygen depletion [49].

Humans living at high altitude also have advantages that facilitate adaptation to hypoxia. Highlanders showed a significant elevation of glucose preference when compared to lowlanders [52]. In addition, long term residents of high altitude exhibited increased angiogenesis and red blood cells overproduction that enhance oxygen transport. Unfortunately, it also represents a maladaptation since detrimental consequences such as pulmonary hypertension and increased blood viscosity are common among highlanders. Tibetan natives exposed to high altitudes for more than 30,000 years are believed to have bypassed such maladaptation. They harbor a gain-of-function mutation in the prolyl hydroxylase domain 2 (PHD2 or EGNL1) and hypoxia-inducible factor 2α (HIF2A or EPAS1) haplotypes that confer less sensitivity to hypoxia and increased shift to anaerobic metabolism, respectively [53].

### 4.2. Metabolic Acidosis Buffering Systems

The strategy to avoid lactic acidosis is diverse. Turtles are assumed to buffer acidosis by releasing calcium carbonate from the shell. Accordingly, hatchlings that have uncompleted shell development are less tolerant to anoxia than adults [5]. Crucian carp can convert anaerobically lactate into ethanol, which then diffuses out over the gills into the water [58] and some cetaceans have shown amino acid changes in genes of enzymes involved in gluconeogenesis, which are expected to increase the ability of lactate removal after diving [50]. On the contrary, *Drosophila* and locust suffer acidification of the haemolymph during anoxia and up to date no strategies of lactate buffering have been described [27,31]. In humans, lactate concentration has been documented to rise during ischemia. The potential role of this metabolite as a biomarker for ischemic stroke has been studied and, unlike previous studies suggesting a detrimental effect, recent reports showed that lactate accumulation protects neurons in vivo [59].

### 4.3. Strategies to Control Excitotoxicity

The loss of ionic homeostasis after ATP depletion does not occur in tolerant species, which survive hypoxia without apparent neuronal damage. Pond turtle and crucian carp brains have been reported to undergo channel arrest but also suppress neuronal activity by a mechanism that involves increased γ-Aminobutyric acid (GABA) release as well as upregulated number of GABA receptors [60]. Nevertheless, other authors studying brain transcriptomic response of the Western Painted Turtle did not observe any change in genes related to ion channels or synaptic transmission, which were expected to be overexpressed [61]. In addition, the high density of δ-opioid (DOR) receptors in the brain may also protect turtles from glutamate excitotoxicity [62].

NMR and artic ground squirrel are believed to reduce synaptic activity by regulating glutamate receptors to reduce calcium ions permeability and therefore prevent excitotoxicity [60]. Accordingly, εPKC was found to delay neuronal depolarization during ischemia in artic ground squirrels by inhibiting both Na^+^/K^+^-ATPase and voltage-gated sodium channels [43]. In *Drosophila*, chronic depolarization in anoxia also results in a loss of excitability that allows the initiation of the paralytic state. Flies does not regulate extracellular ions concentration but are able to tolerate extracellular ionic disruption [30].

Tolerance of diving mammals can be explained, in part, by intrinsic neuronal properties. Cortical slices from deep-diving hooded seals showed a significantly lower rate of hypoxic depolarization compared to adult mice [51]. Hence, stabilizing ion gradients during hypoxia is a protective strategy in different animal species.

### 4.4. Enhancement of the Antioxidant Defense

Hypoxia tolerant species have different complementary ways to protect themselves from the oxidative damage. Pond turtles present constitutively high levels of antioxidants and neuroglobin, an oxygen binding haem protein found in the CNS, which represent a pre-conditioning state upon hypoxia-reoxygenation exposure [63]. On the contrary, other animals transiently upregulate antioxidant defenses to fight oxidative stress during hypoxia or reoxygenation. The European common frog (*Rana temporaria)* exhibits lower oxidative metabolism during brumation than during peak metabolic activity while other species, like the carp and the goldfish, present higher levels of antioxidants during brumation than when active to protect against oxidative damage associated with the arousal-associated reperfusion [64].

*Drosophila* has shown to respond differently when exposed to intermittent or constant hypoxia. Constant hypoxia modifies the expression of a greater number of genes when compared to intermittent hypoxia. Among them, Heat Shock Proteins (HSPs) are the most upregulated gene family [32]. HSP are responsible for maintaining protein conformation by either repairing or degrading them, a mechanism that protects cells from stress. Specifically, Hsp70 and Hsp23 showed to significantly participate in animal survival after hypoxia. On the other hand, intermittent hypoxia mainly involves genes related to neurotransmitter transport and defense response [32].

NMR cortical neurons maintained nitric oxide homeostasis during hypoxia to avoid a burst upon reoxygenation while mice cortical slices exhibited increased production of nitric oxide upon reperfusion [65]. Moreover, NMR retain robust mitochondrial membrane integrity compared to mice, allowing for a better coupling efficiency of the electron transport chain and less free radical generation [55], which can partially explain physiological differences between both rodent species in hypoxia resistance and cellular dysfunction following reoxygenation.

Despite the fact that some of these molecular responses are also induced in humans when exposed to hypoxia, they are obviously not enough to protect against reoxygenation damage. Moreover, Tibetans, who are adapted to live in chronic hypoxic environment, also present increased levels of ROS and oxidized lipoproteins [54].

## 5. Preclinical Proof of Concept

In biomedical research, species that are closer to humans, such as non-human primates, are usually considered the best models to study human pathology. However, the Nobel laureate discoveries on the molecular mechanisms of circadian rhythm using the fruit fly and the identification of the oxygen sensor HIF-prolyl hydroxylases in *C. elegans* highlight the usefulness of other model systems. Since oxygen is necessary for all aerobic organisms, species surviving to long periods of hypoxia could reveal novel mechanisms of adaptation. Despite the hypoxia response being highly conserved, tolerant species are evolutionary different and comparative models from evolutionary biology could provide unique opportunities to identify distinctive features, as has been highlighted recently [66]. In fact, after many years of research and scientific progress we are still lacking a medical treatment for ischemic brain damage. However, evolution found the solution for neuronal resilience to hypoxia in several animal species. The fact that all these mechanisms are different and complementary to each other makes them more attractive for the use of combination therapies, something that is very new in the field of neuroprotection.

**Figure 3:**
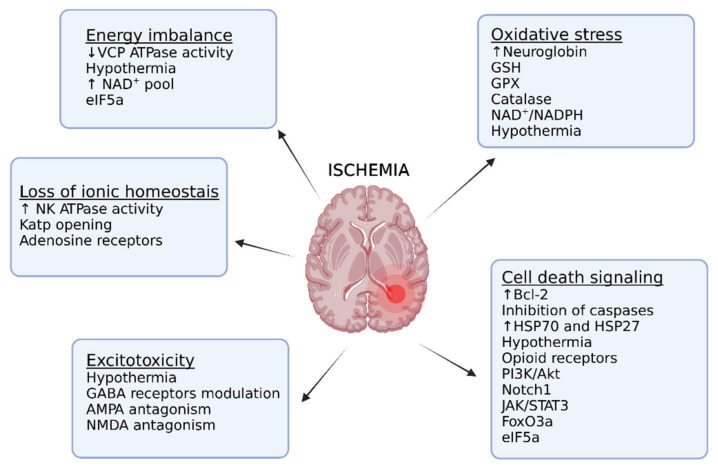
Mechanisms involved in hypoxia tolerance of translational potential for stroke treatment. Abbreviations: VCP ATPase: valosin-containing protein (VCP) ATPase; NAD^+^:nicotinamide adenine dinucleotide; eIF5A: eukaryotic initiation factor 5A: N/K ATPase: Na^+^/K^+^-ATPase; Katp channel: ATP sensitive potassium channel; GABA: Gamma aminobutyric acid; *NMDA*: N-methyl-d-aspartate; GSH: glutathione; GPx: glutathione peroxidase; NADP: Nicotinamide adenine dinucleotide phosphate; Bcl-2: B-cell lymphoma; HSP: heat shock protein: PI3K: Phosphoinositide 3-kinase; Akt: protein kinase B; Notch1: Neurogenic locus notch homolog protein 1; JAK: Janus kinase; STAT3: signal transducer and activator of transcription 3; FoxO3a Forkhead Box O3a.

Hypoxia tolerance results from the simultaneous regulation of several molecular mechanisms and many of them have been tested to work in animal models of cerebrovascular disease, as depicted in Figure 3. Many preclinical studies in rodents have reported neuroprotective mechanisms for stroke that are also taking part in the coordinated response of hypoxia tolerance in several species and are summarized in Table 2. Eukaryotic initiation factor 5A (eIF5A) and Forkhead box O 3 (FoxO3) are both interesting pathways involved in hypoxia tolerance and are discussed in more detail here.

### 5.1. Eukaryotic Initiation Factor 5A (eIF5A)

The role of the eukaryotic initiation factor 5A (eIF5A) on regulating hypoxia was first reported in *Drosophila* during long-term hypoxia exposure [113]. eIF5A is a very conserved transcription factor involved not only in translation but also in several cellular functions such as autophagy, cell growth and proliferation. In eukaryotes, two isoforms of this factor are known, which are encoded by different genes. eIF5A-1 isoform is expressed ubiquitously whereas the eIF5A-2 protein is restricted to testis and brain, but it is also present in certain cancer tissues and it is considered as an oncogene. A unique feature of eIF5a is that it is the only family of cellular proteins that contains the aminoacid hypusine, which is formed by two sequential reactions: (1) the conjugation of the aminobutyl moiety of spermidine to a specific lysine residue and (2) its hydroxylation, catalysed by deoxyhypusine synthase (DHS) and deoxyhypusine hydroxylase (DOHH), respectively [114]. Hypusination is essential for eIF5A activation and has been described to play a role in mitochondrial respiration by controlling the expression of several proteins involved in oxidative phosphorylation and the tricarboxylic acid cycle [115]. Accordingly, acute inhibition of this pathway by *N*1-guanyl-1,7-diaminoheptane (GC7), a DHS inhibitor, induced a metabolic shift toward glycolysis and mitochondrial remodelling while downregulated respiratory chain complexes activity to promote anoxic cell tolerance. Administration of GC7 improved hypoxia tolerance of flies fed on a polyamine supplemented diet by a DHS dependent mechanism [113]. GC7 treatment was also described to reduce oxygen consumption in vitro and to improve transplant output using a preclinical pig kidney transplant model by protecting against ischemia/reperfusion–induced renal injury [116]. Recently, inhibition of eIF5A hypusination pathway was tested as a potential target for stroke. In vitro, GC7 protected primary neurons from oxygen-glucose deprivation and glutamate excitotoxicity-induced cell death. GC7 was able to preserve the mitochondrial membrane potential, preventing ROS generation. In addition, a single intraperitoneal administration of the DHS inhibitor either 2 h before or 2 h after the occlusion of the middle cerebral artery reduced the infarcted area and promoted motor and cognitive recovery in mice [67].

The clinical application of hypusination of eIF5A may have a more restricted effect that would be expected for a protein controlling translation since it is important to control only a subset of the total population of mRNAs. Pharmacological approaches to suppress eIF5A hypusination include DHS inhibitors, such as GC7, Semapimod (CNI1493) or deoxyspergualin (gusperimus); inhibitors of the ornithine decarboxylase, which is the first enzyme in the polyamine pathway and suppresses the synthesis of the spermine serving as substrate for DHS, like difluoromethylornithine (DFMO); and also DOHH inhibitors (ciclopirox, deferiprone, and mimosine). In addition, other modifications of eIF5A, such as acetylation, can affect the intracellular distribution of the protein and impede the hypusination of the post-translational modified protein [117]. GC7 has not been tested in humans yet, but a drug with reported DHS inhibitor activity, Semapimod, was safely administered in clinical trials [118]. However, Semapimod showed a lower inhibitory effect and targeted also other cellular pathways including cytokine production, Toll-like receptor and MAP Kinase activation, which may cause additional off-target effects [119]. It is the same case for deoxyspergualin [120]. DFMO was also reported to be well tolerated and was developed to treat African sleeping sickness [121]. Then, clinical applications of hypusination inhibitors is feasible and the recent preclinical data on ischemic models provide a potential therapeutic target for ischemia/reperfusion related diseases.

### 5.2. Forkhead Box O 3 (FoxO3)

The Forkhead box O (FOXO) is one subfamily of the fork head transcription factor (FOX) family involved in cell fate decisions, such as proliferation and survival. Mammalian FOXO protein family share the characteristic of being regulated by the insulin/PI3K/Akt signaling pathway and consist of FoxO1, FoxO3, FoxO4, and FoxO6. Among them, FoxO3a has been broadly studied because of its implication in several diseases. FoxO3a transcriptional activity is mainly regulated by phosphorylation, which prevents the nuclear shuttle and therefore impedes its transcriptional activity. Phosphorylated FoXO3a remains at the cytoplasm and is ubiquitinated by an E3 ligase for proteasomal degradation [122]. FoxO3a is expressed in all tissues of the body, and it seems to be conserved throughout evolution. Previous studies reported that induction of DAP-16, the mammalian homolog of FoxO3a, plays a role in hypoxia tolerance in *C. elegans* [123] and other member of the family, FOXO1, regulates the transcription of antioxidant enzymes during dehydration/rehydration stress in frogs (similar to ischemia/reperfusion) [124]. In *Drosophila*, FOXO transcriptional activity is induced in larvae and adults during hypoxia and its activation is required for hypoxia tolerance in flies. The authors showed that the suppression of Akt signalling during hypoxia induced FOXO transcriptional activity and, consequently, increased flies’ survival after hypoxia exposure mediated by the immune transcription factor Relish (NFκB). Moreover, changes in FOXO activation altered glucose metabolism during hypoxia [125]. Similarly, FOXO participates in the ability of turtle brains to survive anoxia/reoxygenation damage by preventing oxidative damage [126].

During experimental stroke, FOXO3 activation is increased in the penumbra area and functions as a protective mechanism against ischemic injury. FOXO3 viral overexpression induced autophagy in the brain and reduced the infarcted area after stroke in rats [68]. This effect has also been reported in other models of ischemia. Circular RNA Foxo3, which interacts with Foxo3 proteins and inhibits the phosphorylation of Foxo3, is involved ischemia-reperfusion injury occurring in heart transplantation in mice [127]. Accordingly, Epigallocatechin-3-gallate, a green tea polyphenol that modulates FOXO transcription factors, improved long term recovery after stroke in mice and promoted neurogenesis [128]. Additionally, the protective effect against ischemic brain damage of Salvianolic acid A, a compound extracted from traditional Chinese medicine *Salvia miltiorrhiza* Bunge, is partially attributed to the modulation of AKT/FOXO3a/BIM pathway [129].

### 5.3. Other Mechanisms Involved in Hypoxia Tolerance

Hypoxia-inducible factor 1α (HIF-1α) is considered the master transcriptional regulator of the coordinated response to hypoxia and has been the focus of much research. Many strategies have been tested to work in preclinical models by stabilizing HIF-1α via HIF-prolyl hydroxylases (PHDs) inhibition or through other mechanisms [130]. HIF-1α regulates several processes including erythropoiesis, cell proliferation and energy metabolism, and it has been shown that ischemic tolerance is partially mediated by HIF-1α induction [131]. In hypoxia susceptible animals HIF-1α expression during ischemia is biphasic and, depending on the time-point, it can lead to apoptosis (early activation) or promote cell survival (late activation) [132].

Contrary to the ability of tolerant animals to adapt their cellular energetics during hypoxia, in the course of stroke brain cells cannot cope with the dramatic decrease of ATP, so it seems reasonable that lowering ATP consumption may help to prevent ischemic brain damage. In this line of evidence was developed KUS121, an inhibitor of the ATPase activity of valosin-containing protein (VCP), the most abundant soluble ATPase in mammalian cells including neurons. VCP participates in many cellular processes such as proteostasis or cell cycle progression and KUS121 was shown to behave as an ATP regulator without apparently affecting other VCP cellular functions [133]. In vivo administration of KUS121 improved functional deficits and reduced brain infarction volume after transient focal cerebral ischemia [72]. KUS121 also reduced the ischemic damage and improved best-corrected visual acuity in a small group of patients with non-arteritic central retinal artery occlusion [134].

Hypothermia can also to help to maintain ATP levels by lowering cerebral metabolic rate Consistently, moderate hypothermia is the standard care for perinatal hypoxic-ischemic encephalopathy [135]. In a meta-analysis, the use of hypothermia in animal models of ischemic stroke was shown to reduce infarct volumes by 44% on average with the highest efficacy achieved cooling to lower temperatures, treating before or at the onset of ischemia, and using transient instead of permanent ischemia animal models [136]. Unfortunately, whole body cooling is not an easy procedure for bigger organisms because it is usually invasive and requires patient sedation and intubation. Then, selective brain hypothermia has become an option to avoid systemic side effects. Local endovascular infusion (LEVI) was safely implemented in patients undergoing ischemic stroke, but the neuroprotective efficacy remains to be established in a clinical setting [136].

Hypothermia affects a wide range of cellular processes so the reduction of energy demand is not the only mechanism that can explain its therapeutic effects. Physical and pharmacological-induced hypothermia in mice were published to downregulate proinflammatory cytokines expression and microglial activation after experimental stroke [81]. Furthermore, lowering body temperature has shown to prevent apoptosis [137], reduce anoxic depolarization and protect from BBB breakdown^68^.

When ATP level falls below 80%, intracellular ATP-dependent activities, such as protein folding, proteasomal degradation and autophagy, terminate or slow down. Protein misfolding, aggregation and destruction of organelles contribute to hypoxic-ischemic brain injury pathology [138]. HSP is a family of molecular chaperones synthesized in response to stress that participate in proteostasis. It has been shown that overexpression of Hsp70 and Hsp27 plays a part in ischemic tolerance induced by preconditioning [139]. Different studies reported reduced apoptotic and infarcted area in ischemic brains after viral overexpression or recombinant protein administration of both Hsp70 [87,89] and Hsp27 [90,91,140]. Moreover, administration of Hsp90 ligands, which results in increased transcription of chaperones, ameliorated brain edema, inflammation and neurobehavioral deficits after experimental models of subarachnoid hemorrhage (SAH) [141] as well as MCAO [88].

Interestingly, the neuroprotective effect of Hsp27 against ischemia-reperfusion injury is dependent on posttranslational modifications of the protein by phosphorylation [91,140]. Neuroprotective effects of HSP27 have been largely attributed to its antiapoptotic activity but specific HSP27 overexpression in endothelial reduced actin polymerization and the disassembly of junctional proteins in cerebral endothelial cells and it was sufficient to attenuate BBB disruption and provide long lasting protection after stroke in vivo [142].

Trehalose, a nonreducing sugar present in insects’ hemolymph, has been also proposed to be neuroprotective by playing a role in protein stabilization and autophagy. Trehalose is not synthesized or stored in vertebrates, but it can be hydrolyzed by a specific enzyme and it has been suggested that the neuroprotective effects require the gastrointestinal system processing [143]. Oral administration of trehalose has shown neurological benefits in a mouse model of tauopathy, reversing the dropout of dopamine neurons mediated by the induction of autophagy [144]. In vitro, trehalose accumulation protected cells against low oxygen stress [145]. In vivo administration of trehalose after experimental cardiac infarction protected against ischemia-induced cardiac remodeling by inducing autophagy [146] but the effects on experimental ischemic stroke have not been reported yet.

The extraordinary capacity of hypoxia tolerant animals of attenuating excitotoxicity by the modulation of ion channels is being targeted by different therapeutic approaches. In the first place, activation of GABA receptors, whom inhibitory signaling is impaired after stroke, reduced infarct size and improved recovery after ischemia in rodents and prevented parietal cortex damage after permanent Middle Cerebral Artery Occlusion (MCAO) in primates [147]. Different GABA agonists, such as chlormethiazole or diazepam, have been studied in clinical trials but no convincing evidence was obtained to support their use for treating neither acute ischemic nor haemorrhagic stroke. Moreover, the sedative effects of GABAergic drugs hamper their use [148]. Additionally, research indicates that the initial acute phase of hyperexcitability is followed by delayed inhibitory neurotransmission resulting in neuronal hypoexcitability so the therapeutic effects could be limited by a short time window [149] and, consistently, post-stroke administration of drugs increasing GABA signalling were shown to improve motor function [93].

Neuron hyperexcitability has been also addressed by blocking excitatory receptors. As occurred with GABAergic drugs, NMDAR antagonists resulted in neuroprotection in preclinical studies, but the direct blockade of NMDARs has failed for clinical use. NMDARs activation can result in both pro-survival or excitotoxic effects depending on whether the receptors are intra or extrasynaptically located and the subunits that constitute them. Since neuronal death can be modulated by downstream non canonical pathways independent to Ca^2^+ entry, then, the research now focuses on disrupting the pathways downstream the receptor without blocking it [150]. Accordingly, the peptide GluN2BCT1292–1304 protected neurons against ischemic damage in vivo by blocking the formation of the DAPK1-Glut2B complex [151]. A critical NMDA downstream target during ischemia is pannexin-1 (Panx-1). It has been described that NMDA overstimulation during ischemia activates the Src family kinases to open Panx1 channels during anoxic depolarization [152]. NMDARs, Src kinase and Panx1 form a signaling complex activated by ligand binding to NMDARs and not by ion conductance and the disruption of this signalsome reduced the infarct volume in vivo [100]. In addition, Tat-K13, which prevents NMDAR-induced PTEN nuclear translocation, also reduced ischemic brain damage in rats [99]. Several drugs have been designed to uncouple GluN2B-PSD95-nNOS complex. After ischemia, PSD-95 behaves as a scaffold protein to recruit nNOS to the NMDAR complex, leading a more effective nNOS activation and generation of the cytotoxic compound NO [153]. Tat-NR2B9c (Nerinetide) and NA-1 peptide reduced infarct volume and improved neurobehavioral outcomes when administered after tMCAO in rats [98], reduced infarct volumes and preserved neurological function at 30 days in primates [154] and reduced the number and volume of strokes and improved neurological outcome in patients undergoing endovascular intracranial aneurysm repair [155]. As stated above, absolute benefit was described for Nerinetide in stroke patients who did not receive alteplase.

Similarly, the small molecule ZL006 showed neuroprotective effects in vitro and reduced cerebral ischemic injury in both mouse and rat stroke models [150]. Since the blockade of AMPA receptors can reduce secondary NMDA activation several antagonists have been tested for ischemia. AMPAR antagonists were reported to work when administered after tMCAO in vivo and the therapeutic effects persisted for 7 days after ischemia [66,97]. Cerestat and Selfotel, two non-competitive AMPA antagonists underwent phase III clinical trials but they were prematurely terminated because of safety concerns [156].

Ionic homeostasis in the turtle brain during anoxia is partially mediated by adenosine receptor and Katp channels [38]. Extracellular adenosine increases soon after ischemia in turtles and in human brains after reperfusion. Adenosine possesses a high binding affinity to the Adenosine Receptor A1 receptor (A1R), mainly localized in the CNS, and activation of which reduces neuronal excitability by preventing Ca^2+^ influx and glutamate release. On the other hand, binding to the widely distributed Adenosine 2A receptor (A2AR) increases calcium entrance [157]. Systemic administration of adenosine is not a suitable therapy for stroke since it is quickly degraded and has cardiovascular deleterious effects. However, adenosine infusion into the ischemic striatum ameliorated neurological outcome and infarct volume after transient focal cerebral ischemia in rats [158]. A1R and A2AR ligands have a dual role depending on their administration regime. In the acute phase after stroke, activation of A1R resulted in neuroprotection whereas chronic treatment worsened neuronal loss because of receptor desensitization [159]. In contrast, early A2AR antagonism provide control excessive excitotoxicity, and delayed A2AR agonism provide protracted protection by controlling massive blood cell infiltration in the hours and days after ischemia [157].

Finally, a shared strategy among almost all tolerant species to avoid reoxygenation damage is to increase their antioxidant defenses, and the same approach seems to work in sensitive animals. Antioxidant systems include the activity of the main antioxidant enzymes: superoxide dismutase (SOD), glutathione peroxidase (GPx), and catalase (CAT); as well as the non-enzymatic defenses glutathione (GSH) and vitamins A, C and E [160]. The transcription factor Nrf2 is the major coordinator of antioxidant genes activation. Preclinical studies support the beneficial effects of Nrf2 activation both before and after the occlusion in transient and permanent MCAO models [161] and overexpression of SOD, GPx and CAT were independently reported to protect mice from ischemic stroke [110,111]. Administration of GSH after MCAO in rats significantly reduced infarct volume and BBB disruption [109]. Moreover, the evidence of transgenic mice with reduced GPx support the importance of oxidative stress for ischemic injury [109]. Common to these antioxidants is the requirement of NADPH and NAD+ as cofactors. Exogenous NAD+ prevented ATP decrease and lactate accumulation in mice brains after stroke. It reduced the ischemic brain damage and edema and neurological outcome. Moreover, the combination of NAD+ and NADPH extended the therapeutic window of NAD+, enhanced the protective effect and suppressed cell death signaling pathways [112].

Despite preclinical success of exogenous antioxidants, tirilazad and NXY-059, two examples of radical scavengers tested in humans, have failed to achieve the primary end-point of neuroprotection in humans [162]. A possible reason may be that these drugs work to reduce ROS damage but do limit their generation or interfere with other pathological events upstream of the ischemic cascade.

## 6. Conclusions and Future Directions

Many drugs have been preclinically tested and hundreds of them reached clinical trials, but up to date there is no single approved drug apart from tPA/TNK that can preserve brain function after stroke. Neurons from other animal species can survive the lack of oxygen but the complexity that human brain has achieved during evolution make us more vulnerable to the energetic failure during ischemia. This review summarizes the current knowledge on the mechanisms that allow for brain hypoxia tolerance of the main resistant species, including several mammals such as diving mammals and the naked mole rat. There are common physiological mechanisms for all the tolerant species, such as the reduction on the energy demand or a metabolic switch to the glycolytic pathway, but the molecular changes taking place during hypoxic stress are not fully depicted. Although most of the hypoxia tolerant species are not routinely used for biomedical research, their value as model organisms for ischemia is undisputable. When studying human samples or experimental models of stroke the simultaneous activation of protective and pathological pathways makes difficult to discriminate if molecular alterations are beneficial or harmful for recovery. However, hypoxia resistant species have developed strategies to suppress those pathological pathways and boost protective mechanisms. Then, as more information become available from tolerant species we will be closer to outline the molecular signature of hypoxia tolerance.

FoxO3 and eIF5A are just two recent examples of how data obtained from tolerant species can turn about into potential treatments for stroke, at the moment in the experimental setting only. Most of the mechanism already described were also found to be successful in experimental studies and include, as reviewed, molecular processes allowing for the modulation of cellular energetics, constitutively improved antioxidant defense systems and strategies to reduce excitotoxic damage that lead to molecular mechanisms of cell death.

The therapeutic efficacy of some of the protective mechanisms involved in hypoxia tolerance has been widely studied in mammalian models. This is the case of downregulating basal metabolism using hypothermia. Whole-body hypothermia reduced the risk of death or disability in child with moderate or severe hypoxic-ischemic encephalopathy [163]. Moreover, the innate protective mechanism of ischemic preconditioning, which has been investigated for heart and brain ischemia, shares several features with anoxia/ischemia tolerance, including mitochondrial ROS generation, Ca^2+^ signalling and apoptosis inhibition [164]. The information provided from tolerant species, adapted through millions of years of evolution, may contribute to provide new therapeutic options by clarifying the multifactorial mechanism involved in hypoxia tolerance.

Considering the heterogeneity of stroke pathology and the multiplicity of mechanisms involved that are differently regulated over the time, one possible reason for the failure on treatment translation is the use of monotherapeutic strategy. By combining several molecular mechanisms described to play a role in hypoxia tolerance, one can probably recreate what happens in a tolerant brain during hypoxia. This approach can ultimately facilitate the development of new therapies that would not be based on neuroprotection hypothesis but on proven natural brain resistance. However, the clinical reality is very complex and designing these combinations is not an easy task.

It is likely that some drug combinations have additive effects based on the independent actions of the individual drugs while other combinations may provide synergic effects. The degree of additive or synergic effect is time consuming and very difficult to evaluate using experimental models, and in vitro system do not adequately represent the complexity of the disease. Therefore, in silico modeling of stroke using system biology becomes a very useful tool. These mathematical models enable us to make predictions about the effects of combining two or more drugs to achieve a good outcome, as well as to apply internal filters to avoid adverse effects or administration incompatibilities. In this sense, another logical approach would be to evaluate the possibility of administration at different time points over the course of the disease.

Therefore, further studies elucidating hypoxia resistance mechanism of tolerant species would guarantee new molecular targets for neuroprotective therapies that, combined into pharmacological cocktails represent a more realistic approach to control the complex pathogenesis of stroke.

## Figures and Tables

**Table 1:** Mechanisms contributing to hypoxia resistance described for different tolerant species. Abbreviations: ADP: adenosine diphosphate; Akt: protein kinase B; ATP: adenosine triphosphate; AR: adenosine receptors; Bax: Bcl2 Associated X protein; Bcl-2: B-cell lymphoma; DOR: delta-opioid receptors; EGLN1: Egl-9 Family Hypoxia Inducible Factor 1; EPAS1 (Endothelial PAS Domain Protein 1); FBP1: Fructose-1,6-bisphosphatase; FOXO: Forkhead box O 3; GABAR: Gamma aminobutyric acid (GABA) receptor; GAPDH: Glyceraldehyde-3-Phosphate Dehydrogenase; GLUT5: Glucose transporter 5; GPI: Glucose-6-phosphate isomerase; Hg: haemoglobin; (HIF-1α): Hypoxia-inducible factor 1α; HSP: heat shock protein; JAK: Janus kinase; Katp: ATP sensitive potassium channel; KHK: ketohexokinase; LDHA: lactate dehydrogenase A; LDHD: lactate dehydrogenase D; Mgb: myoglobin; NKA: Na^+^/K^+^-ATPase Ngb: neuroglobin; NMDAR: N-methyl-d-aspartate (NMDA) receptor; NO: nitric oxide; NT: neurotransmitter; PI3K: Phosphoinositide 3-kinase; Akt: protein kinase B; PC: pyruvate carboxylase; PCK1: phosphoenolpyruvate carboxykinase 1; PDCD6IP: Programmed Cell Death 6 Interacting Protein; PDE1β: pyruvate dehydrogenase E1 component subunit beta; PDHc: pyruvate dehydrogenase; STAT3: signal transducer and activator of transcription 3; TCA: tricarboxylic acid cycle; VCP: valosin-containing protein.

		Specie	Type of Resistance	Metabolism	Source of Energy	Neurotransmission	Molecular Mechanisms of Neuroprotection	pH Buffering
Invertebrates		**Locust** L. migratoria Ref: [27,28,29]	Natural: environmental O_2_ depletion Experimental anoxia: 4 h at 30 °C	Reversible coma ↓ ATP muscle ↑ K ↓ Na haemolymph ↓ haemolymph volume	Glycogen ↓ ATP		PTPN1 and PDHE1β variants in Tibetan locust and increased PDHE1β expresion : enhanced aerobic metabolism	↓ hemolymph pH
		**Fruit fly** D. melanogaster Ref: [30,31,32]	Natural: environmental O_2_ depletion Experimental anoxia: LT_50_ = 8 h Selected resistant flies: live under constant 4 % O_2_	Reversible coma ↓ metabolism ↓ ATP ↑ K Hypoxia resistant flies: ↓ Glycolytic, TCA and β-oxidation enzymes and respiratory complexes	Glycogen Trehalose	Editing of ion channels by adenosine deaminase improves hypoxia survival	Threalose, tsp1 Polyols, β-alanine, taurine ↑ HIF1α ↑ FOXO Acute: ↑ Hsp70, Hsc73 Chronic: ↑ Notch, Toll, hairy	↓ haemolymph pH ↑ lactate, alanine, succinate and acetate
Vertebrates		**Frog** R. sylvatica and amurensis Ref: [33,34]	Natural: Freeze during the winter (−2.5 °C) Experimental anoxia: 24 h at 5 °C	↓ ATP ↑ glycogenolysis: ↑ glucose but ↓ lower products	Glycogen		Changes in phosphoproteins to use glucose and urea as cryoprotectant ↑ Glycerol Low succinate/fumarate ratio	↑ lactate, alanine, succinate
		**Crucian carp** C. carassius Ref: [4,35,36,37]	Natural: 4–5 months’ anoxia under frozen lakes Experimental anoxia: 7 days at 8 and 13 °C. 6 weeks at 2 °C	↓ heart rate ↓ GAPDH, LDH Ethanol as end product Increased brain blood flow Suspend some sensory functions	Glycogen stored in liver High affinity Haemoglobin	↓ Glutamate and aspartate ↑ GABA and Glycine	Hypothermia p53R2 paralogs Hypothermia ↓ Ca influx	Lactate transported to muscle and converted to ethanol by PDHc
		**Pond turtle** T.Scripta C.Picta Ref: [4,38,39,40,41,42]	Months of anoxia during hibernation Experimental: 30 hours at 20 °C 7 weeks at 3 °C	Reversible coma ↓ energy demand ↓ proteins involved in ATP supply ↓ heart rate and heat production Low balanced ATP levels Channel arrest	Brain glycogen stores ↑ glycolysis ↓ protein synthesis Constant ADP in heart	↑ GABAR and AR ↓ excitatory NT and NMDAR activity Katp channel activation ↑ DOR Check for signal arousal	Hypothermia ↑ Bcl2: Bax ↓ PDCD6IP ↑ PI3K, AKT, ERK, p53 ↑ HIFα ↑ JAK/STAT in liver Antioxidant defense preconditioning: antioxidants, HSP, adenosine, Ngb and succinate/fumarate ratio ↓ VCP, GAPDH	Lactate is buffered by calcium carbonate realease from the shell
	Mammals	**Artic ground squirrel** U.Paryii Ref: [5,43,44,45]	Natural: hypoxemia during arousal from hibenation and euthermy Experimental: 8 min anoxia (euthermic)	↓ metabolism up to 1–2% of basal when hibernating	Lipid metabolism	↓ NMDAR ↓ excitotoxicity (↓ NR1 subunit) ↓ NKA activity	↑ antioxidant defense↓ NKA and Na channelsHypothermiaImmunosuppression, Anticoagulant blood↑ HIF1α	Good pH bufferingArousal: ↑ lactate
		**Naked mole rat** H.glaber Ref: [46,47,48]	Natural: chronic hypoxia living in burrows Experimental anoxia: 18 min at 33 °C 6 min at 37 °C	Awake and active during hypoxia Loss of consciousness with sporadic breathing during anoxia ↓ metabolism ↓ heart and respiratory rate	Hypoxia:↑ glycolysis Anoxia: fuelled by fructose	Maintenance of synaptic transmission NMDAR isoforms less permeable to calcium	↑ GLUT5 mRNA and protein levels ↑ KHK-A KHK-C ↓ intracellular calcium Maintained mitochondrial membrane integrity	No signs of acidosis Cardiac function facilitates lactate clearance
		**Diving mammals** Whales, seals Ref: [49,50,51]	Behavioural pattern (diving) at 3–4 °C	↓ metabolism in some organs Anaerobic metabolism (lactate at the end of the dive) ↓ heart rate	Large glycogen stores Oxygen in Hg, Mgb and Ngb and splenic contraction but critical at the end of the dive	Maintain spiking in vitro Reconfiguration of neuronal activity ↓ NMDA activity ↓ Ca influx	Stress tolerance (antioxidants) Ngb ↑ HIF1α	Positive selection genes in cetaceans (LDHA, LDHD, PC, PCK1, FBP1, and GPI)
		**Human** Ref: [52,53,54]	High altitude adapted Tibetans Ischemic preconditioning	↑ glucose uptake and glycolysis ↓ mitochondrial glucose oxidation ↑ free fatty acids Changes in haemoglobin levels	↑ glucose ↓ lipid metabolism		Mutations in EGNL1 (↑ O2 affinity and ↓ p23 affinity), EPAS (↑ anaerobic shift) and PPARA genes ↑ NO ↑ cerebral perfusion ↑ HSP-27, NOS, GDNF, VEGFa, TGF β1, LIF and TIMP-1 ↓ inflammation (↓ MCP1) and coagulation	↑ lactate

**Table 2:** Preclinical studies supporting the effectiveness of targeting the protective mechanisms described in tolerant species for the treatment of experimental stroke. Abbreviations: AR1: adenosine receptor 1: AR2: adenosine receptor 2; BBB: blood brain barrier; DFO: deferoxamine; EPO: erythropoietin; ET1: endothelin 1; i.c.v.: intracerebroventricular; i.p.: intraperitoneal; i.v.: intravenous; i.n.: intranasal; Ngb: neuroglobin; pMCAO: permanent middle cerebral artery occlusion; p.o.: per os (oral); s.c.: subcutaneous; tMCAO: transient middle cerebral artery occlusion; VEGF: Vascular Endothelial Growth Factor. ↓: decrease; ↑ increase.

Target	Treatment	Species	Regime	Stroke Model	Outcome	Time
eIF5a	GC7 Ref. [67]	Mice C57BL/6	2 h before MCAO (i.p,) 2 h after MCAO (i.p,)	tMCAO for 60 min	↓ infarct volume ↑ neurological recovery	24 h and 4 days
FOXO3a	Viral over-expression Ref. [68]	Rat Sprague-Dawley	1 week before MCAO	tMCAO for 2 h	↓ infarct volume	24 h
HIF-1α		Mice nPHD2 KO Ref. [69]		pMCAO	↓ infarct volume ↑ sensory motor function ↑ VEGF	7 and 30 days
	DFO Ref. [70]	Rat Sprague-Dawley	48 h before MCAO at 3 h intervals (i.n.)	pMCAO	↓ infarct volume ↓ neurologic deficit	5 days
	GSK360A Ref. [71]	Rat Sprague-Dawley	18 h and 5 h before MCAO (p.o.)	tMCAO for 2 h	↓ infarct volume ↓ neurological deficit ↑ EPO and VEGF	4 weeks,
Reduction of VCP ATPase activity	KUS121 Ref. [72]	Mice C57BL/6 and B17	After occlusion + after reperfusión	B17: distal tMCAO for 22 min C57BL/6: distal pMCAO + 30 min hypoxia (10%O_2_)	B17: ↓ infarction volume and ↑ NeuN C57BL/6: ↓ infarction volume, ↑ rotarod and removal test performance	24 h
Bcl-2 induction	Bcl-2 viral over-expresion Ref. [73]	Rat Sprague-Dawley	14 h before MCAO (Cortical infusion)	Distal tMCAO for 3 h Distal pMCAO	=infarct volume ↓ neuronal death per infected cell ↓ cytochrome C release ↓ Caspase-3	48 h
	PACAP Ref. [74]	Mice C57BL/6 and PACAP −/−	1 h after MCAO (i.c.v./i.v.)	pMCAO	↓ infarct volume ↑ functional recovery (NSS score) ↓ cytochrome C release	24 h
	PACAP deficiency Ref. [75]	Mice PACAP +/− (C57BL/6)		tMCAO for 12 min	↑ neurogenesis	7 days
PI3K/Akt activation	Melatonin Ref. [76]	Mice C57BL/6	Right after reperfusion (i.p.)	tMCAO for 30 or 90 min	↓ infarct volume ↑ BBB integrity	24 and 72 h
	Electo Acupuncture Ref. [77]	Rat Sprague-Dawley	24 h after MCAO (30 min/day for 3 days)	tMCAO for 2 h	↓ infarct volume ↓ caspase-3 ↓ autophagy	72 h
	FGF10 Ref. [78]	Mice C57BL/6	30 min before MCAO (i.c.v.)	tMCAO for 2 h	↓ infarcted area↓ neurological deficit ↓ TUNEL^+^ cells, Caspase-3, 8 and 9 ↓ TNFα, IL6	24 h
Caspase-6 inhibition	Z-VEID-FMK Ref. [79]	Rat	0 h and 24 h after reperfusion (i.v.)	thromboembolic focal cerebral ischemia	↓ infarct volume and brain edema ↓ neurological deficit (Benderson´s) ↓ caspase-3, 6 and 8 ↑ prolifeRat ing cells	48 h and 7 days
Caspase-8 inhibition	Z-IETD-FMK Ref. [79]	Rat	0 h and 24 h after reperfusion (i.v.)	thromboembolic focal cerebral ischemia	↓ infarct volume ↓ edema ↓ neurological deficit (Benderson´s) ↓ caspase-3, 6 and 8 ↑ prolifeRat ing cells	48 h and 7 days
Caspase-3 inhibition	Z-DEVD Ref. [80]	Rat Sprague-Dawley	5 h after MCAO (i.v.)	tMCAO for 2 h	↓ infarction range ↓ caspase-3 and apoptosis ↓ neurological deficit (Benderson´s)	48 h
Therapeutic hypothermia	HSP-201 (NTR1 agonist) Ref. [81]	Mice C57BL/6	30 min after MCAO plus repeated doses (i.p.) for constant 33 °C 6 h	Distal pMCAO	↓ infarct volume ↓ edema ↑ neurological performance (corner and cylinder tests) ↓ TNFα, IL1β, MCP-1, IBA-1 ↑ IL10 ↓ M1 ↑ M2	6, 24 and 72 h
Notch1 activity	DAPT/DBZ (γ-secretase inhibitors) Ref. [82]	Mice C57BL/6	30 min before occlusion/ 4 h after/6 h after	tMCAO for 1 h	↓ infarct volume ↑ neurological performance	3 h
	Notch1Tg Antisense Ref [82]	Mice NAS Tg (C57BL/6)		tMCAO for 1 h	↓ infarct volume ↑ neurological performance	3 h
	Simvastatin (↑ activity) Ref [83]	Rat Wistar	24 h before MCAO (daily, 7 days, p.o.)	tMCAO for 2 h	↑ arterial density ↑ vascular cell prolifeRat ion	14 days
JAK/STAT3 inhibition	STAT3 siRNA Ref. [84]	Rat Adult hypertensive	1 h before MCAO (i.c.v.)	tMCAO for 1 h	↓ infarct volume ↓ TUNEL^+^ cells ↓ neurological deficit	24 h
	AG490 (JAK inhibitor) Ref. [84]	Rat Adult hypertensive	24 h before MCAO (i.c.v. infusion)	tMCAO for 1 h	↓ infarct volume ↓ TUNEL^+^ cells ↓ neurological deficit	24 h
	SMND-309 (JAK/STAT activator) Ref. [85]	Rat Sprague-Dawley	9 h after reperfusion (i.v.)	tMCAO for 90 min	↓ infarct volume ↑ functional recovery (NSS score) ↑ EPO ↓ vascular permeability	7 and 14 days
Na^+^/K^+^ ATPase activity	↓ NKAα1 activity Ref. [86]	Mice NKAα1 +/− (C57BL/6)		tMCAO for 1 h	↑ infarct volume	24 h
	DR-Ab (↑ activity) Ref. [86]	Mice C57BL/6	1 h before/1 h after MCAO (i.c.v.)	tMCAO for 1 h	↓ infarct volume	24 h
HSP-70	rHSP70 Ref. [87]	Rat Wistar	20 min before or 2 h after MCAO (i.v.)/s.c. alginate	tMCAO for 45 min	↓ infarct volume	48 and 72 h
	17-DMAG (HSP90 inhibitor) Ref. [88]	Mice C57BL/6	7 days before ischemia every other day (p.o.)	tMCAO for 1 h	↓ infarct volume ↓ neurological deficit ↓ IBA1, MHCII, NFκB, TNFα, IL1β, ICAM1, iNOS	24 h
	Hsp70 viral over- expresión Ref. [89]	Rat Sprague-Dawley	12 h before and 0.5, 1 and 2 h after ischemia (i.c.v.)	ICA occlusion for 1 h	Profilactic: ↑ neuronal survival Therapeutic: =infarct size	48 h
HSP27	hHSP27 Ref. [90]	Mice C57BL/6	1 h after reperfusion (i.v.)	tMCAO for 1 h	↓ infarct volume ↓ neurological deficit ↓ apoptosis ↓ oxidative DNA damage, lipid peroxidation and glial activation	24 and 72 h
	prHSP27 Ref. [91]	Mice C57BL/6	2 h after reperfusion (i.v.)	tMCAO for 1 h + D-glucose for hemorrhagic transformation	↓ infarct volume and edema ↓ BBB permeability ↓ MMP9 ↓ neurological severity ↓ mortality and hemorrhagic transformation	24 h
	HSP27 overexpression Ref. [92]	Mice Hsp27 Transgenic C57BL/6		tMCAO for 1 h	↓ infarct volume ↓ Behavioural deficit ↓ BBB permeability and apoptosis in microvessel walls ↓ Brain water content ↓ Neuropil infiltRat ion and	24 and 96 h
GABA receptor modulation	L-655,708 (GABA inverse agonist)	Rat Sprague-Dawley Ref. [93]	7 days before stroke for 2 weeks (s.c.)	Intracortical ET1	↓ infarct volume ↓ neurological severity	3 weeks
		Mice C57BL/6 Ref. [94]	3 days after stroke (i.v. minipump)	Photothrombosis	↑ functional recovery	7 days
Adenosine receptor modulation	AR1 KO Ref. [95]	Mice AR1 −/− (C57BL/6)		Bilateral common artery occlusion	=neuronal damage	4 days
	8-CPT (AR1 antagonist) Ref. [95]	Mice C57BL/6	30 min before ischemia (i.p.)	Bilateral common artery occlusion	↑ neuronal damage	4 days
	AR2 KO Ref. [96]	Mice AR2 −/− (C57BL/6)		tMCAO for 2 h	↓ infarct volume ↓ neurological deficit score ↓ brain water content and glutamate	2, 22 and 26 h
AMPA receptor antagonism	Perampanel Ref. [66]	Rat Sprague-Dawley	After reperfusion (i.p.)	tMCAO for 90 min	↓ infarct volume and edema ↓ inflammation (IBA1, TNF-α, IL1β) ↓ oxidative stress ↓ Bax ↑ Akt ↑ Bcl-XL	7 days
	YM872 Ref. [97]	Rat Sprague-Dawley	2, 3 or 4 h after reperfusion (i.v.)	tMCAO for 3 h	↓ infarct volume ↓ neurological deficit	24 h and 7 days
Reduction of NMDA receptor activation	Tat-NR2B9c (NA-1) Ref. [98]	Rat Sprague-Dawley	45 min before MCAO (i.v.)	tMCAO for 90 min	↓ infarct volume	24 h
	Tat-K13 Ref. [99]	Rat Sprague-Dawley	2 or 6 h after MCAO (i.v.)	tMCAO for 90 min	↓ infarct volume ↓ neurological deficit (grip and swimming test)	28 days
	TAT-Panx_308_ Ref. [100]	Rat Sprague-Dawley	30 min before or 2 h after MCAO (i.p.)	tMCAO for 1 h	↓ infarct volume ↓ motor deficit	2 and 14 weeks
δ-Opioid receptor agonism	Tan-67 Ref. [101]	Rat Sprague-Dawley	12, 24 or 48 h before MCAO (i.v.)	pMCAO	↓ infarct volume ↑ functional outcome	24 h
	DADLE Ref. [101]	Rat Sprague-Dawley	30 min before MCAO (i.p.)	tMCAO for 2 h	↓ infarct volume ↓ apoptosis	72 h
Katp opening	Diazoxide Ref. [102]	Rat Wistar	24 h before MCAO (i.p.)	tMCAO for 90 min	↓ infarct volume ↓ neurological score (six different tests)	72 h
	BMS-191095 Ref. [103]	Rat Wistar	24 h before MCAO (i.c.v.)	tMCAO for 90 min	↓ infarct volume No effect when administered 30 min before	72 h
	Kir 6.2 over-expression Ref. [104]	Mice Kir 6.2 Tg (C57BL/6)		pMCAO + systemic hypoxia (8% O_2_ for 20 min)	↓ cortical infarct	72 h
	Kir 6.1 Ref. [105]	Mice Kir 6.1 +/− (C57BL/6)		tMCAO for 1 h	↑ Infarct size and neuronal loss ↑ Neurological deficit and ↑ ECM degeneRat ion and ER stress	24 h
Ngb over- expression	Ngb transgene Ref. [106]	Mice Ngb-Tg2 (C57BL/6)		pMCAO	↓ Infarct area	24 h
	Ngb viral over- expression	Rat Sprague-Dawley Ref. [106]	3 weeks before MCAO (i.c.v.)	tMCAO for 90 min	↓ infarct volume ↓ Neurological deficit (NSS score)	24 h
		Rat Wistar Ref. [107]	14 days before ischemia	tGCI for 10 min	↑ Cell survival in the CA1 area ↑ Atpb1 expression and Na/K ATPase activity	7 days
	TAT-mNgb Ref. [108]	Mice C57BL/6	2 h before the occlusion (i.v.)	tMCAO for 2 h	↓ infarct volume ↓ Neurological deficit	24 h
Modulation of the antioxidant defense system	GSH infusion Ref. [109]	Rat Sprague-Dawley	10 min after MCAO (i.v.)	tMCAO for 1 h	↓ infarct volume ↓ ROS, Foxo-3 ↑ Bcl-2, PI3K/Akt	48 h
	GPX viral delivery Ref: [110]	Rat Sprague-Dawley	12 h before/2 or 5 h after MCAO	tMCAO for 1 h	↑ Neuronal survival ↓ Apoptosis, cytochrome C release	24 h
	Catalase transgene Ref. [111]	Mice CAT-Tg (C57BL/6)		tMCAO for 40 min	↑ infarct volume	48 h
	NAD +/− NADPH Ref. [112]	Mice ICR	0 h after reperfusion (i.v.)	tMCAO for 2 h	↓ infarct volume	28 days
